# Mushroom poisoning deaths and prevention practices in Hunan, 2014–2023

**DOI:** 10.1371/journal.pone.0326107

**Published:** 2025-06-16

**Authors:** Shilan Wu, Qiang Wang, Hongbo Duan, Jiahao Xiong, Tianbing Lai, Chune Wang, Jinjun Liang, Huayun Jia

**Affiliations:** Hunan Provincial Center for Disease Control and Prevention (Hunan Academy of Preventive Medicine), Changsha, Hunan, PR China; Lusofona University of Humanities and Technologies: Universidade Lusofona de Humanidades e Tecnologias, PORTUGAL

## Abstract

**Background:**

Wild mushroom poisoning is a major cause of foodborne illness-related deaths in Hunan Province. By analyzing the epidemiological characteristics of fatal incidents due to wild mushroom poisoning in Hunan Province over the past decade and preventive measures implemented in recent years, in this study, we aimed to provide insights for nationwide prevention and control of wild mushroom poisoning, focusing on reducing mortality.

**Methods:**

Data from the “Foodborne Disease Outbreak Surveillance System” in Hunan Province were used to describe the characteristics of wild mushroom poisoning. Kernel density analysis was performed using ArcMap 10.8.1 to identify spatial clustering.

**Results:**

It showed that from 2014 to 2023, 80 fatal wild mushroom poisoning incidents and 111 deaths, with a case fatality rate of 1.5%, were reported in Hunan Province. June (40 events, 50.0%) and August (17 events, 21.3%) are the peak periods for wild mushroom poisoning. The species most frequently associated with fatal poisoning were *Russula subnigricans* (20 events, 25.0%), *Amanita fuliginea* (18 events, 22.5%), and *Amanita rimosa* (11 events, 13.5%). Kernel density analysis indicated that the fatalities were primarily concentrated in the central and eastern regions of Hunan Province. All (100%) fatal incidence collected from households with poisoning caused by accidental harvesting and consumption. Rural areas accounted for 93.8% of the fatal incidents. The highest proportion of deaths (48.7%) occurred in the age group of 60 years and above.

**Conclusion:**

Fatalities due to wild mushroom poisoning incidents exhibit seasonal and regional variations. We call for multi-sectoral collaboration in prevention and control efforts, focusing on key populations, regions, and time to conduct public education and promotion and ensure smooth access to medical care, which are crucial strategies for preventing wild mushroom poisoning incidents and reducing mortality.

## Introduction

Wild mushrooms are highly diverse and widely distributed, with approximately 14,000 species documented globally, including over 4,000 species identified in China, of which 660 are toxic [1,2]. Edible wild mushrooms are valued not only for their palatability but also for their rich protein, carbohydrate, and essential mineral contents. Additionally, they exhibit potential bioactive properties, including immune enhancement, lipid regulation, and antitumor activity, making them nutritionally and medicinally significant [3]. However, inadvertent collection and consumption of toxic wild mushrooms can result in severe illness and even death [4].

The extensive variety of poisonous mushroom species and their diverse toxin profiles lead to markedly different incubation periods (ranging from 30 minutes to several days) and a broad spectrum of clinical manifestations. These may include gastrointestinal symptoms (vomiting, diarrhea, nausea), neuropsychiatric effects (hallucinations), and in severe cases, life-threatening hepatorenal damage that may progress to multi-organ failure [5]. In China, mushroom poisoning manifests with distinct clinical syndromes depending on the fungal species involved, and is currently categorized into principal types: hepatotoxic, nephrotoxic, hemolytic, rhabdomyolytic, gastrointestinal, neurotoxic, and photosensitivity dermatitis types, with additional miscellaneous presentations also observed [6]. Cases involving highly (deadly) toxic mushrooms impose a significant health burden on affected families as there is no specific antidote for mushroom poisoning. Mushroom poisoning has become a major cause of morbidity and mortality in foodborne diseases globally [7–9]. In this study, we aimed to examine the fatalities associated with wild mushroom poisoning in Hunan Province over the past decade and outline prevention and control strategies that may serve as a reference for other regions.

## Methods

The data for this study were sourced from the “Foodborne Disease Outbreak Surveillance System” in Hunan, which encompasses one provincial, 14 municipal, and 122 county-level Centers for Disease Control and Prevention (CDC). According to the CDC, any foodborne disease outbreaks involving two or more individuals or resulting in at least one death must be reported. The data sources included foodborne disease outbreaks identified by healthcare institutions during routine clinical practice, detected through surveillance systems and investigated by CDCs at all levels, and reported following food safety incident investigations conducted by food safety regulatory agencies. This analysis included outbreaks diagnosed as wild mushroom poisoning that resulted in fatalities based on epidemiological investigations, history of wild mushroom consumption, and clinical manifestations between 2014 and 2023.

A database was constructed using Excel (version 2019, Microsoft, USA), the data were checked and summarized. Epidemiological characteristics were systematically examined through descriptive statistical methods, with all categorical variables presented as frequency counts and corresponding percentages (%). The fundamental principle of kernel density analysis is that geographical events are more likely to occur in regions with higher spatial point density and less likely in areas with lower point density [10]. This analytical approach enables efficient identification of disease case clusters and it was performed using ArcMap (version 10.8.1, ESRI, USA) to explore spatial clustering. The base map for the analysis was sourced from the web (Guihuayun.com, Access data: July 12, 2024) with map review number GS Jing (2022) No. 1061.

All data were collected from January 1, 2014, to December 31, 2023. This retrospective study using anonymized data was exempt from ethical approval in accordance with institutional policies for retrospective research. Additionally, all analyses were conducted following standardized protocols to ensure consistency and reliability across the dataset.

## Results

From 2014 to 2023, Hunan Province documented 2,254 wild mushroom poisoning incidents, accounting for 51.4% (2,254/4,386) of all reported foodborne disease outbreaks. These incidents involved 7,326 patients, among which 80 discrete poisoning events led to 111 fatalities, representing 81.0% (111/137) of total foodborne disease-related deaths during this period. The overall case fatality rate stood at 1.5%. Since 2014, the number of fatalities initially increased and then declined (Table 1).

**Table 1:** Wild mushroom poisoning in Hunan Province (2014–2023).

Year	Total incidents	Number of cases	Fatal incidents	Number of deaths	Case Fatality Rate%
**2014**	22	92	4	8	8.7
**2015**	119	474	7	11	2.3
**2016**	134	502	8	10	2.0
**2017**	321	1064	20	31	2.9
**2018**	289	998	7	10	1.0
**2019**	220	751	9	12	1.6
**2020**	574	1739	9	11	0.6
**2021**	200	624	8	8	1.3
**2022**	115	379	4	6	1.6
**2023**	260	703	4	4	0.6
**Total**	2,254	7326	80	111	1.5

**Fig 1:**
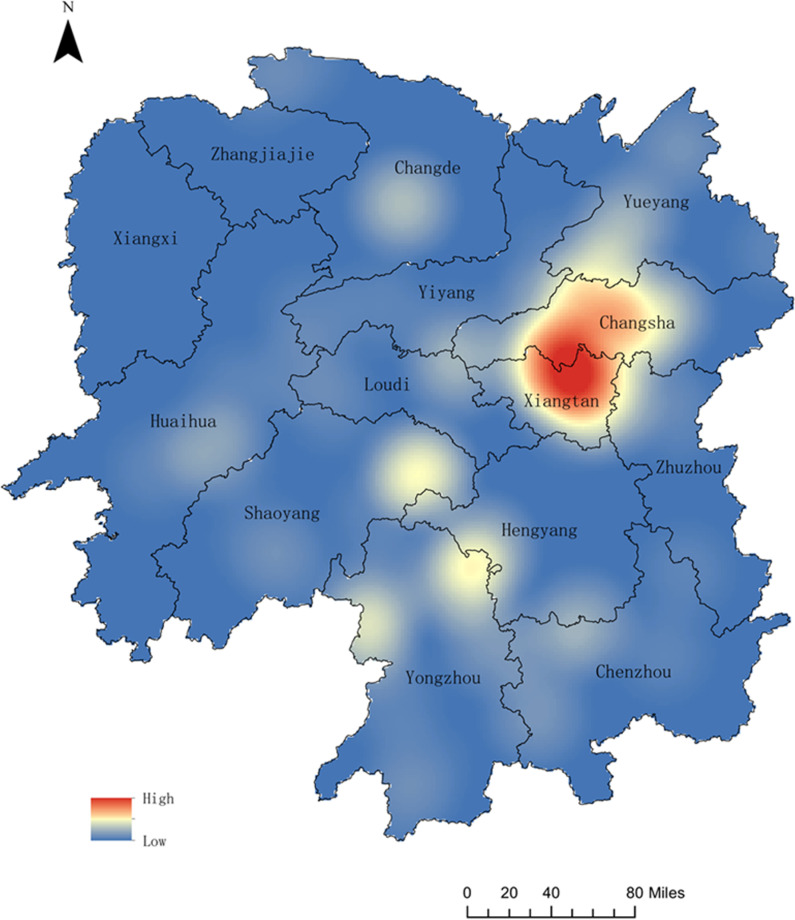
Spatial distribution of wild mushroom poisoning fatalities in Hunan Province. The map displays the results of kernel density estimation. Red areas indicate high clustering intensity, with darker shades representing higher density values. Conversely, blue areas represent low clustering intensity, where case occurrences are sparser.

**Fig 2:**
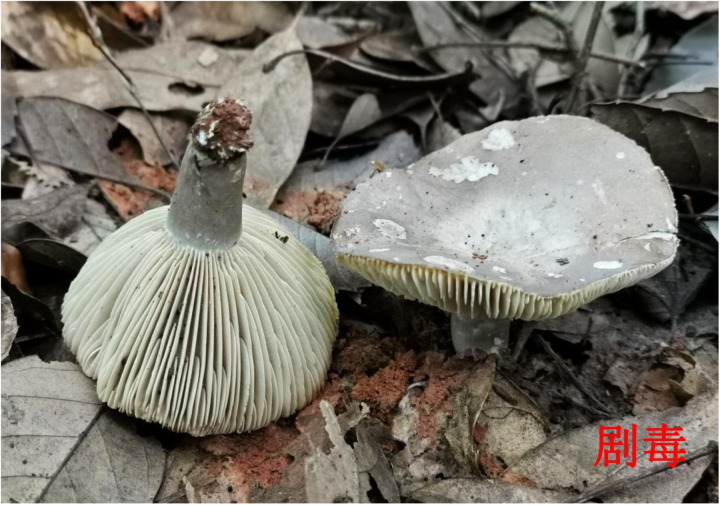
Morphology of “*Russula subnigricans”* (lethally toxic). Image source: Atlas of Common Poisonous Mushrooms in Hunan, Hunan CDC [11]. The original image was cropped for clarity.

**Fig 3:**
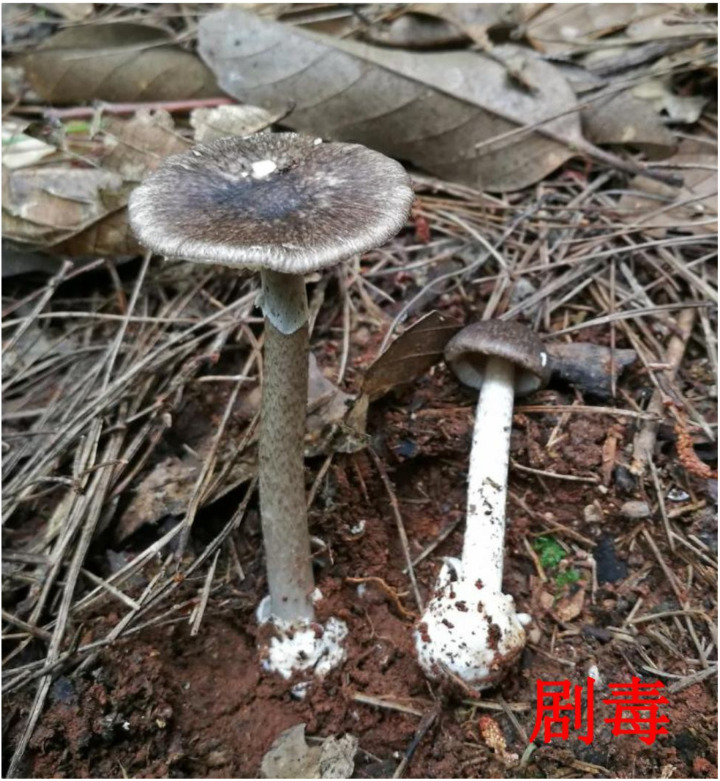
Morphology of “*Amanita fuliginea”* (lethally toxic) **.** Image source: Atlas of Common Poisonous Mushrooms in Hunan, Hunan CDC [11]. The original image was cropped for clarity.

**Fig 4:**
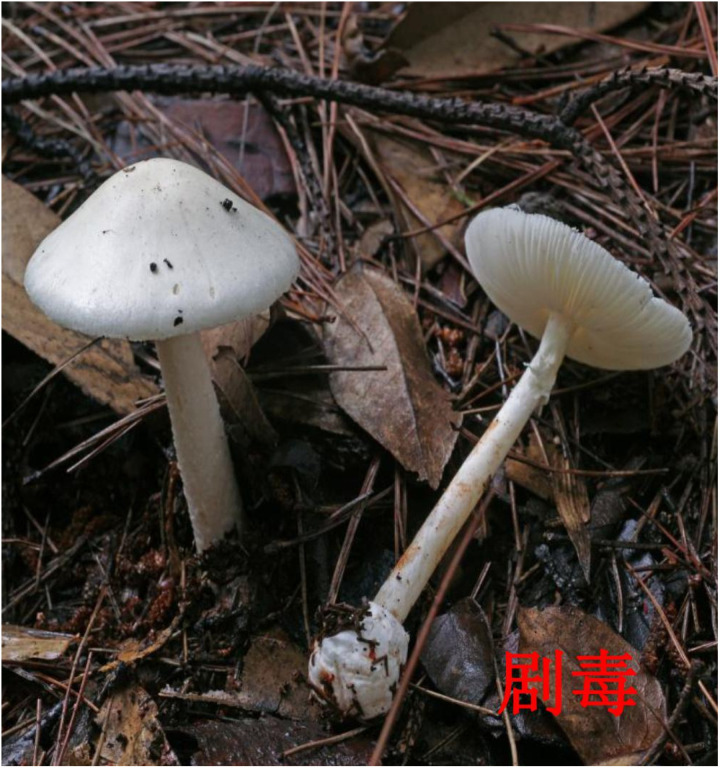
Morphology of “*Amanita rimosa”* (lethally toxic). Image source: Atlas of Common Poisonous Mushrooms in Hunan, Hunan CDC [11]. The original image was cropped for clarity.

Fatal mushroom poisoning incidents occurred between April and November, with the highest in June (40 events, 50.0%), followed by August (17 events, 21.3%). All 12 municipalities in Hunan Province reported fatal incidents, with the highest numbers in Changsha (14 events, 44 deaths) and Xiangtan (13 events, 44 deaths). Kernel density analysis indicated that the fatalities (number of deaths) were primarily concentrated in the central and eastern regions of Hunan Province. (Fig 1). Most deaths occurred in rural areas (75 events, 93.8%). In addition, The highest mortality was observed in individuals aged 60 years and above (54 deaths, 48.7%). All cases involved mushrooms collected from households with poisoning caused by accidental harvesting and consumption. The species most frequently associated with fatal poisoning were *Russula subnigricans* (20 events, 25.0%), *Amanita fuliginea* (18 events, 22.5%), and *Amanita rimosa* (11 events, 13.5%). The morphological features of these species are illustrated in Figs 2–4. Moreover, the highest number of deaths was associated with *A. fuliginea* ingestion (31deaths, 27.9%). See Table 2.

**Table 2:** Fatalities due to wild mushroom Poisoning.

Group		Events	Illnesses	Hospitalization	Deaths
		Number(%)	Number(%)	Number(%)	Number(%)
**Month**	**April**	1(1.3)	1(0.4)	1(0.5)	1(0.9)
	**May**	4(5)	12(4.9)	11(5.7)	6(5.4)
	**June**	40(50)	125(51.2)	120(61.9)	62(55.9)
	**July**	6(7.5)	18(7.4)	12(6.2)	6(5.4)
	**August**	17(21.3)	47(19.3)	30(15.5)	23(20.7)
	**September**	10(12.5)	35(14.3)	19(9.8)	11(9.9)
	**October**	1(1.3)	3(1.2)	0(0)	1(0.9)
	**November**	1(1.3)	3(1.2)	1(0.5)	1(0.9)
**Municipal**	**Changsha**	14(17.5)	44(18)	34(17.5)	21(18.9)
	**Xiangtan**	13(16.3)	44(18)	35(18)	20(18)
	**Yongzhou**	13(16.3)	35(14.3)	29(15)	17(15.3)
	**Yueyang**	8(10)	21(8.6)	20(10.3)	10(9)
	**Shaoyang**	7(8.8)	24(9.8)	21(10.8)	10(9)
	**Chenzhou**	6(7.5)	21(8.6)	20(10.3)	8(7.2)
	**Huaihua**	6(7.5)	16(6.6)	11(5.7)	6(5.4)
	**Changde**	3(3.8)	11(4.5)	9(4.6)	6(5.4)
	**Loudi**	3(3.8)	5(2.1)	1(0.5)	4(3.6)
	**Yingyang**	3(3.8)	11(4.5)	2(1)	3(2.7)
	**Hengyang**	2(2.5)	6(2.5)	6(3.1)	4(3.6)
	**Zhuzhou**	2(2.5)	6(2.5)	6(3.1)	2(1.8)
**Area**	**Rural**	75(93.8)	234(95.9)	187(96.4)	105(94.6)
	**Urban**	5(6.3)	10(4.1)	7(3.6)	6(5.4)
**Age**	**0-6 years**	/	19(7.8)	13(6.7)	8(7.2)
	**7-19 years**	/	19(7.8)	14(7.2)	3(2.7)
	**20-59 years**	/	113(46.3)	92(47.4)	46(41.4)
	**60 years and above**	/	93(38.1)	75(38.7)	54(48.7)
**Type of mushroom**	***A fuliginea***	18(22.5)	67(27.5)	65(33.5)	31(27.9)
	***R. subnigricans***	20(25)	59(24.2)	31(16)	21(18.9)
	***A.rimosa***	11(13.8)	29(11.9)	29(15)	13(11.7)
	***Galerina sulciceps***	2(2.5)	4(1.6)	2(1)	2(1.8)
	***Lepiota brunneoincarnata***	1(1.3)	2(0.8)	0(0)	2(1.8)
	***Amanita***	4(5)	7(2.9)	7(3.6)	6(5.4)
	**Mixed**	2(2.5)	14(5.7)	11(5.7)	6(5.4)
	**Unknown**	22(27.5)	62(25.4)	49(25.3)	30(27)
**Total**		80(100)	244(100)	194(100)	111(100)

Different species of highly toxic mushrooms exhibit distinct distribution patterns. Among the known mushroom species involved in fatal incidents, *A. fuliginea* and *A. rimosa* were primarily responsible for deaths in June, whereas *R. subnigricans* was more prevalent in August and surrounding months. The predominant lethal mushroom species varies by region. For example, in Xiangtan, most fatalities were caused by *A. fuliginea*, whereas in Changsha, *A. rimosa* was the leading cause of death. Of the five fatal incidents that occurred due to mushroom poisoning in urban areas, all known species belonged to the *Amanita* genus. See Table 3.

**Table 3:** Distribution of different wild mushroom species for fatal incidents.

Group		*A.fuliginea*	*R.subnigricans*	*A.rimosa*	*Galerina sulciceps*	*Lepiota brunneoincarnata*	*Amanita*	Mixed	Unknown	Total
**Month**	**April**				1					1
	**May**	1							3	4
	**June**	15	1	10		1	3	1	9	40
	**July**	1	4						1	6
	**August**		9				1	1	6	17
	**September**	1	6	1					2	10
	**October**								1	1
	**November**				1					1
**Municipal**	**Changsha**	2	1	6				1	4	14
	**Xiangtan**	8	3				1		1	13
	**Yongzhou**	2	3				1		7	13
	**Yueyang**	3	3	1					1	8
	**Shaoyang**			2				1	4	7
	**Chenzhou**	1	1				1		3	6
	**Huaihua**		4						2	6
	**Changde**	1	1	1						3
	**Loudi**		2			1				3
	**Yingyang**		2		1					3
	**Hengyang**	1					1			2
	**Zhuzhou**			1	1					2
**Area**	**Rural**	17	20	10	2	1	2	2	21	75
	**Urban**	1		1			2		1	5

## Discussion

In 2011, China established a surveillance network for foodborne disease outbreaks. In 2014, the Hunan Branch of the Food Safety Risk Monitoring Center was established, which led to increased personnel and funding monitoring. The number of reported wild mushroom poisoning incidents increased notably in 2015. After peaking in 2017, fatalities due to wild mushroom poisoning have gradually declined in subsequent years.

Globally, most fatal mushroom poisoning cases are associated with toxic *Amanita* species [12]. In Hunan Province, the mushrooms responsible for the highest number of deaths included *A. fuliginea* (which closely resembles the edible *Termitomyces* species), *R. subnigricans* (similar to the edible *Russula densifolia* and *Russula virescens*), and *A. rimosa*. *A. fuliginea* and *A. rimosa* contain amatoxins, which primarily target the liver. The poisoning typically progresses in four stages: the latent period, acute gastrointestinal phase, deceptive recovery phase, and fulminant hepatic failure [13]. The asymptomatic latent period and misleading recovery phase often lead to delayed treatment, thereby increasing the risk of death. And, *R. subnigricans* contains cycloprop-2-ene carboxylic acid as its primary toxin, which can cause rhabdomyolysis, leading to acute kidney injury, myocardial damage, and death in severe cases [14].

Fatal mushroom poisoning incidents exhibited clear seasonality, with the highest number of deaths occurring in June, followed by August. Additionally, the peak occurrence of poisoning varies by mushroom species, even within the summer months: *A. fuliginea* and *A. rimosa* poisoning is most prevalent in June, while *R. subnigricans* poisoning peaks in August. The incidence of mushroom-related deaths varies across regions. Globally, China, Russia, and Ukraine have the highest mortality rates due to mushroom poisoning [15]. In China, the highest number of fatalities occur in the southwestern region, including Hunan Province [16], where deaths are primarily concentrated in the central and eastern areas, particularly in Changsha and Xiangtan. Furthermore, the predominant lethal mushroom species differed across municipalities within Hunan Province. This suggests the need for regional and time-specific prevention strategies. Establishing localized toxic mushroom samples and clinical databases tailored to the characteristics of each area could enhance the effectiveness of control measures.

All fatal incidents occurred within household settings, with 93.8% of these cases occurring in rural areas and a disproportionately higher number of deaths among older age groups. In rural regions, wild mushrooms are readily accessible. However, villagers, particularly older individuals, often lack the necessary knowledge to identify toxic mushrooms accurately. These villagers may have relied on incorrect traditional methods to distinguish between edible and poisonous species. Symptoms following consumption are often not considered seriously, leading to delays in seeking medical attention. Furthermore, underdeveloped rural economies combined with limited diagnostic capabilities and insufficient medical resources may hinder the timely and effective treatment of poisoning cases. Additionally, the physiological decline associated with aging and the prevalence of chronic diseases in older adults increases the complexity of treatment. Numerous studies have identified older adults as the primary demographic of fatalities due to mushroom poisoning [8,9,17]. These findings underscore the need to focus on high-risk populations, enhance clinical treatment, and integrate medical resources to reduce mortality following poisoning incidents.

In response to the severe wild mushroom poisoning in Hunan Province over the past decade, several efforts have been made to address the specific challenges posed by this issue. This established a joint prevention and control system involving the Provincial Food Safety Office, the Health Commission, the Market Supervision Bureau, and local governments. This collaboration has led to the development of the “Comprehensive Risk Grading and Control Guidelines for Wild Mushroom Poisoning in Hunan Province (2021 edition)”. These guidelines classify the risk of wild mushroom poisoning across different regions, enabling the implementation of targeted prevention and control measures that are dynamically adjusted each year. Additionally, multiple departments collaborated to conduct targeted awareness campaigns, reaching out to grassroots and rural communities to change the villagers’ perceptions.

In terms of public education, emphasis is placed on the key points. In June, the emphasis is on preventing poisoning by highly toxic *Amanita* species by highlighting their distinct morphological characteristics and typical clinical poisoning symptoms. Similarly, in August, we focused on poisoning from *R. subnigricans*. Educational initiatives include posting poisoning alerts on Hunan CDC’s WeChat account and mainstream media, creating illustrated guides on wild mushroom prevention, developing easy-to-understand popular science books [18], producing short educational videos, and designing dialect audio warnings for eight high-risk areas. Additionally, Hunan issued mushroom poisoning alerts via weather forecasts.

Efforts to strengthen the integration of medical and preventive measures have intensified. A WeChat group was established comprising grassroots staff, public health personnel, clinical treatment experts, and mycology identification specialists. When healthcare institutions identify poisoning cases, they promptly report them to the local CDCs for epidemiological investigation, with experts assisting in identifying the mushroom species and guiding the treatment. Furthermore, treatment guidelines have been developed, and medical personnel with corresponding training provided. Each city and prefecture have designated at least one general hospital as a treatment center for highly toxic mushroom poisoning cases, establishing streamlined referral and treatment procedures to enhance post-poisoning care.

As a result of these efforts, the number of deaths from wild mushroom poisoning in Hunan Province is expected to reach a historic low by 2023. Based on risk monitoring and assessment, the practice of wild mushroom poisoning prevention and control has successfully identified key public health issues and implemented effective risk management and communication strategies. This can be considered a successful application of the food safety risk analysis framework, offering a model for controlling other foodborne diseases. However, this study had some limitations. A significant number of incidents involve unidentified mushroom species owing to insufficient samples or difficulties in morphological identification. Although the number of deaths due to wild mushroom poisoning has decreased, there is still scope for improvement in controlling the number of incidents. This suggests that future efforts should focus on refining public education strategies, implementing more precise control measures, and enhancing the quality and effectiveness of prevention efforts to further reduce the incidence and mortality rates due to wild mushroom poisoning.

## Conclusion

Wild mushroom poisoning represents a significant public health concern in Hunan Province, exhibiting distinct seasonal patterns and regional characteristics. Implementing coordinated, multi-sectoral interventions tailored to these epidemiological features can effectively reduce poisoning-related mortality.
